# Identification of substances which regulate activity of corticotropin-releasing factor-producing neurons in the paraventricular nucleus of the hypothalamus

**DOI:** 10.1038/s41598-020-70481-5

**Published:** 2020-08-12

**Authors:** Yasutaka Mukai, Ayako Nagayama, Keiichi Itoi, Akihiro Yamanaka

**Affiliations:** 1 Department of Neuroscience II, Research Institute of Environmental Medicine, Nagoya University, Nagoya, 464-8601 Japan; 2 Department of Neural Regulation, Nagoya University Graduate School of Medicine, Nagoya, 466-8550 Japan; 3 CREST, JST, Honcho, Kawaguchi, Saitama 332-0012 Japan; 4 JSPS Research Fellowship for Young Scientists, Tokyo, 102-0083 Japan; 5 Department of Neuroendocrinology, Graduate School of Medicine, Tohoku University, Sendai, 980-8575 Japan

**Keywords:** Neuroscience, Physiology

## Abstract

The stress response is a physiological system for adapting to various internal and external stimuli. Corticotropin-releasing factor-producing neurons in the paraventricular nucleus of the hypothalamus (PVN-CRF neurons) are known to play an important role in the stress response as initiators of the hypothalamic–pituitary–adrenal axis. However, the mechanism by which activity of PVN-CRF neurons is regulated by other neurons and bioactive substances remains unclear. Here, we developed a screening method using calcium imaging to identify how physiological substances directly affect the activity of PVN-CRF neurons. We used acute brain slices expressing a genetically encoded calcium indicator in PVN-CRF neurons using CRF-Cre recombinase mice and an adeno-associated viral vector under Cre control. PVN-CRF neurons were divided into ventral and dorsal portions. Bath application of candidate substances revealed 12 substances that increased and 3 that decreased intracellular calcium concentrations. Among these substances, angiotensin II and histamine mainly increased calcium in the ventral portion of the PVN-CRF neurons via AT_1_ and H_1_ receptors, respectively. Conversely, carbachol mainly increased calcium in the dorsal portion of the PVN-CRF neurons via both nicotinic and muscarinic acetylcholine receptors. Our method provides a precise and reliable means of evaluating the effect of a substance on PVN-CRF neuronal activity.

## Introduction

Corticotropin-releasing factor (CRF)-producing neurons in the paraventricular nucleus of the hypothalamus (PVN-CRF neurons) are known to have various physiological functions. For example, these neurons play a central role in stress responses through the hypothalamic–pituitary–adrenal axis (HPA-axis)^1^. In the HPA-axis, PVN-CRF neurons release CRF into the median eminence to enhance secretion of adrenocorticotropic hormone (ACTH) from the anterior pituitary into systemic circulation in response to stress. Reaching the adrenal cortex, ACTH induces secretion of glucocorticoids (GCs), such as corticosterone, to initiate various stress responses. Thus, PVN-CRF neurons are thought to serve as initiators of the HPA-axis. Recently, PVN-CRF neurons have also been implicated in the encoding of opposing valence, where increased and decreased neural activity encodes negative and positive valence, respectively^2^. PVN-CRF neurons are also reported to partially co-express vasopressin^3–5^, oxytocin^6^, neurotensin^7^, enkephalin^7^ and cholecystokinin^8^. PVN-CRF neurons are thought to be primarily glutamatergic but also partially GABAergic^9^, they receive inputs from various brain regions such as the nucleus of the solitary tract^10^, and send projections to the median eminence^11^.

In spite of their importance in the stress response, however, it is still unclear how the activity of PVN-CRF neurons is regulated by other neurons and bioactive substances. Researchers have made an effort to identify the regulators of PVN-CRF neurons by assessing markers of activation, such as the early response gene, c-Fos. After administration of a specific substance, the expression of c-Fos in CRF neurons, expression of the CRF-encoding gene (*Crh*) or the plasma concentration of CRF, ACTH or GCs were analyzed^12^. However, it was difficult to distinguish whether these substances directly or indirectly affected the activity of PVN-CRF neurons. Recently, studies using electrophysiological recordings and calcium imaging identified substances that directly regulate the activity of PVN-CRF neurons, such as gamma-aminobutyric acid (GABA) and glutamate^13^, elabela^14^, estrogen^15^, nesfatin-1^16^ and serotonin^17^. Here we developed a screening method using calcium imaging in brain slices to further identify physiological substances that directly affect the activity of PVN-CRF neurons. A genetically encoded calcium indicator fluorescent protein, yellow cameleon-Nano50^18^, was exclusively expressed in PVN-CRF neurons to monitor the intracellular calcium concentration of PVN-CRF neurons on a time scale of minutes to hours in acute brain slices.

Neuronal intracellular calcium concentration ([Ca^2+^]_i_) can be modulated by various physiological substances via both intracellular and extracellular pathways. For example, CRF and urocortin-III (UCN-III), members of the CRF family of peptides, are reported to increase intracellular calcium release from the endoplasmic reticulum^19^, while UCN-III is also reported to promote extracellular calcium influx via voltage-gated P/Q Ca^2+^ channels^20^. In this study, we monitored substance-induced [Ca^2+^]_i_ modulation in PVN-CRF neurons.

## Results

### Confirmation of exclusive expression of yellow cameleon-Nano50 (YC) in PVN-CRF neurons

**Figure 1:**
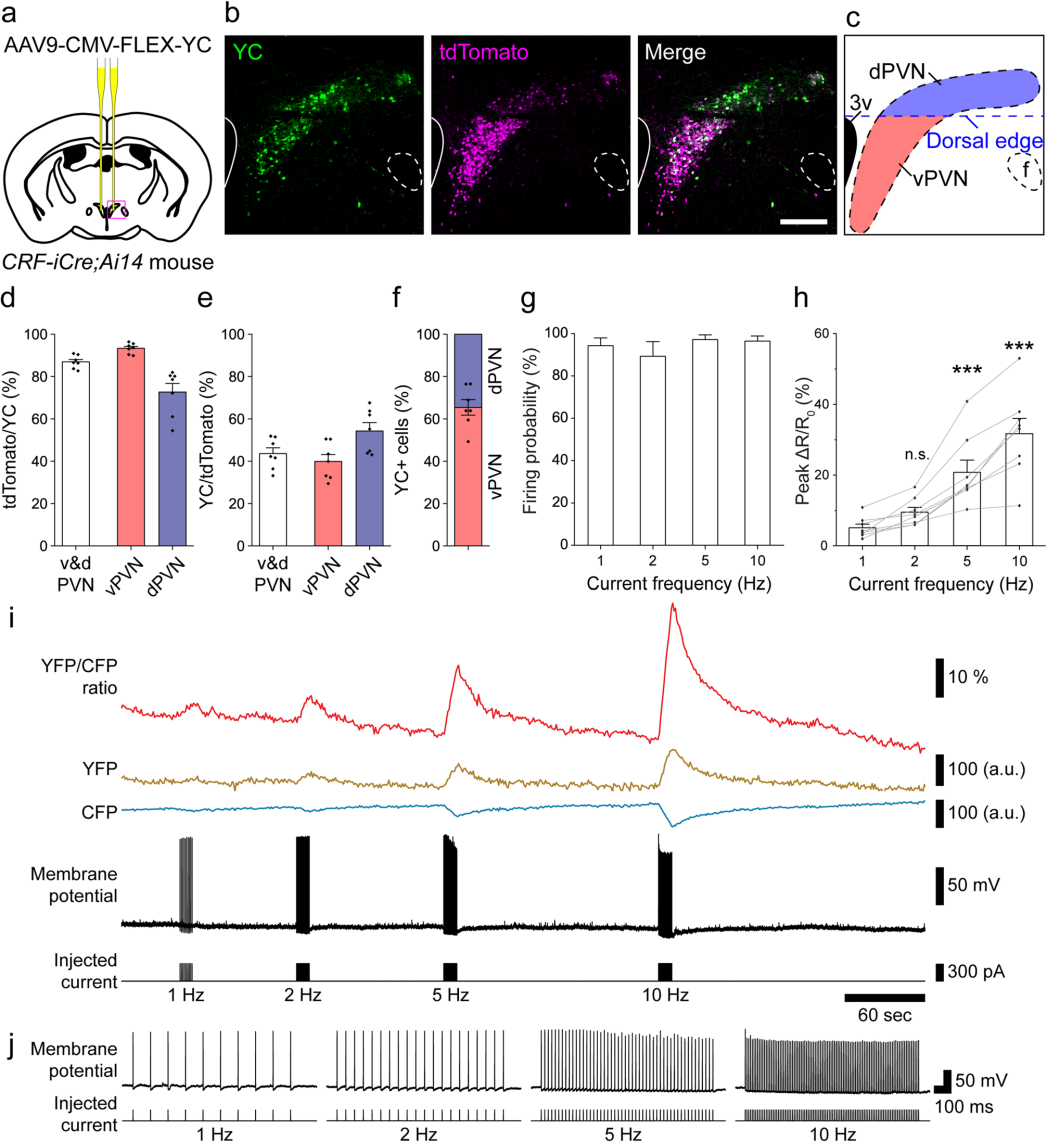
Expression and function of YC in PVN-CRF neurons. (**a**) Injection position of AAV9-CMV-FLEX-YC in the brain. (**b**) Expression of YC in PVN-CRF neurons of *CRF-iCre;Ai14* mice. Green: YC, magenta: tdTomato, scale bar: 200 μm. (**c**) Schematic diagram of the ventral (red) and dorsal (blue) PVN (vPVN and dPVN, respectively) in (**b**). The blue dotted line indicates the mediolateral line through the dorsal edge of the third ventricle which defines the boundary between the vPVN and dPVN. *3v* third ventricle, *f* fornix. (**d**) Percentage of tdTomato-expressing (tdTomato^+^) cells among the YC-expressing (YC^+^) cells in the v&d PVN, vPVN or dPVN, where v&d PVN indicates the entire PVN. (**e**) Percentage of YC^+^ cells among the tdTomato^+^ cells in the v&d PVN, vPVN or dPVN, where v&d PVN indicates the entire PVN. (**f**) Occupancy of YC^+^ cells in the vPVN and dPVN within the entire PVN. (**g**) Probability of action potentials induced by pulse current injection. (**h**) Summary of peak ΔR/R_0_ for pulse current injection-induced action potentials. Statistical analysis was performed using a Kruskal–Wallis test followed by Dunn’s test vs. 1 Hz (***p < 0.001; n.s., not significant). (**i**) Representative trace from simultaneous recording of the calcium signal and membrane potential. Command current was injected through a patch pipette. The frequencies of the injected currents are indicated below the trace. (**j**) Magnified trace of each current injection-induced firing. The frequencies of the injected currents are indicated below the trace. Bar graphs, error bars and dots show the mean value, standard errors and individual data, respectively.

The calcium indicator, YC, was used to monitor intracellular calcium in PVN-CRF neurons. An adeno-associated viral vector (AAV: *AAV9-CMV-FLEX-YC*) which expresses YC in the presence of Cre recombinase was bilaterally injected into the PVN in *CRF-iCre* mice^21^ (Fig. 1a). YC expression in PVN-CRF neurons was confirmed in bigenic mice, *CRF-iCre;Ai14* mice in which Cre expressing CRF neurons exclusively express the red fluorescent protein tdTomato^22^ (tdTomato/YC = 86.9 ± 1.2%; YC/tdTomato = 43.6 ± 2.8%; n = 7 animals; Fig. 1b–e [v&d PVN]). We found strong tdTomato expression in various brain regions, such as the PVN, amygdala and hippocampus, where CRF neurons are already known to be distributed^23^. In the PVN, tdTomato-expressing neurons were found distributed in two regions, as previously reported^24^. tdTomato fluorescence appeared to be high in the ventral portion and low in the dorsal portion of the PVN (Fig. 1b,c). To distinguish the calcium response in the ventral and dorsal portions, we defined a mediolateral line through the dorsal edge of the third ventricle as the boundary between the ventral and dorsal portions of the PVN (vPVN and dPVN, respectively; Fig. 1c). In the vPVN and dPVN, tdTomato expression in YC-expressing cells was 93.3 ± 0.9% and 72.7 ± 4.1%, respectively (Fig. 1d), while YC expression in tdTomato-expressing cells was 39.9 ± 3.3% and 54.3 ± 3.9%, respectively (Fig. 1e). There was a greater proportion of YC-expressing cells in the vPVN than dPVN (vPVN, 65.4 ± 3.6%; dPVN, 34.6 ± 3.6%; n = 7 animals; Fig. 1f).

YC is composed of yellow and cyan fluorescent proteins (YFP and CFP, respectively) fused to a calcium binding domain. When calcium concentrations increase, YFP fluorescence will increase and CFP fluorescence will decrease, and thus the YFP/CFP (Y/C) ratio will increase. To examine the relationship between the YC signal and neuronal activity, we performed simultaneous electrophysiological recordings and calcium imaging in acute brain slices. Positive pulse currents were injected into single cells expressing YC through a recording pipette to evoke action potentials. Calcium imaging in the same neurons showed that the Y/C ratio was increased in a firing frequency-dependent manner. Pulses of 1, 2, 5 and 10 Hz current injection induced firing of 0.95 ± 0.03, 1.81 ± 0.12, 4.88 ± 0.10 and 9.69 ± 0.22 Hz, respectively (Fig. 1g), and induced increases of 5.1 ± 1.0, 9.5 ± 1.4, 20.8 ± 3.5 and 31.7 ± 4.3 ΔR/R_0_ (%), respectively (n = 8 cells, Fig. 1h–j). Thus, these results confirmed that functional YC was exclusively expressed in PVN-CRF neurons and that an increase in the Y/C ratio was correlated with neuronal activity.

### Screening of substances that directly affect activity of PVN-CRF neurons

**Figure 2:**
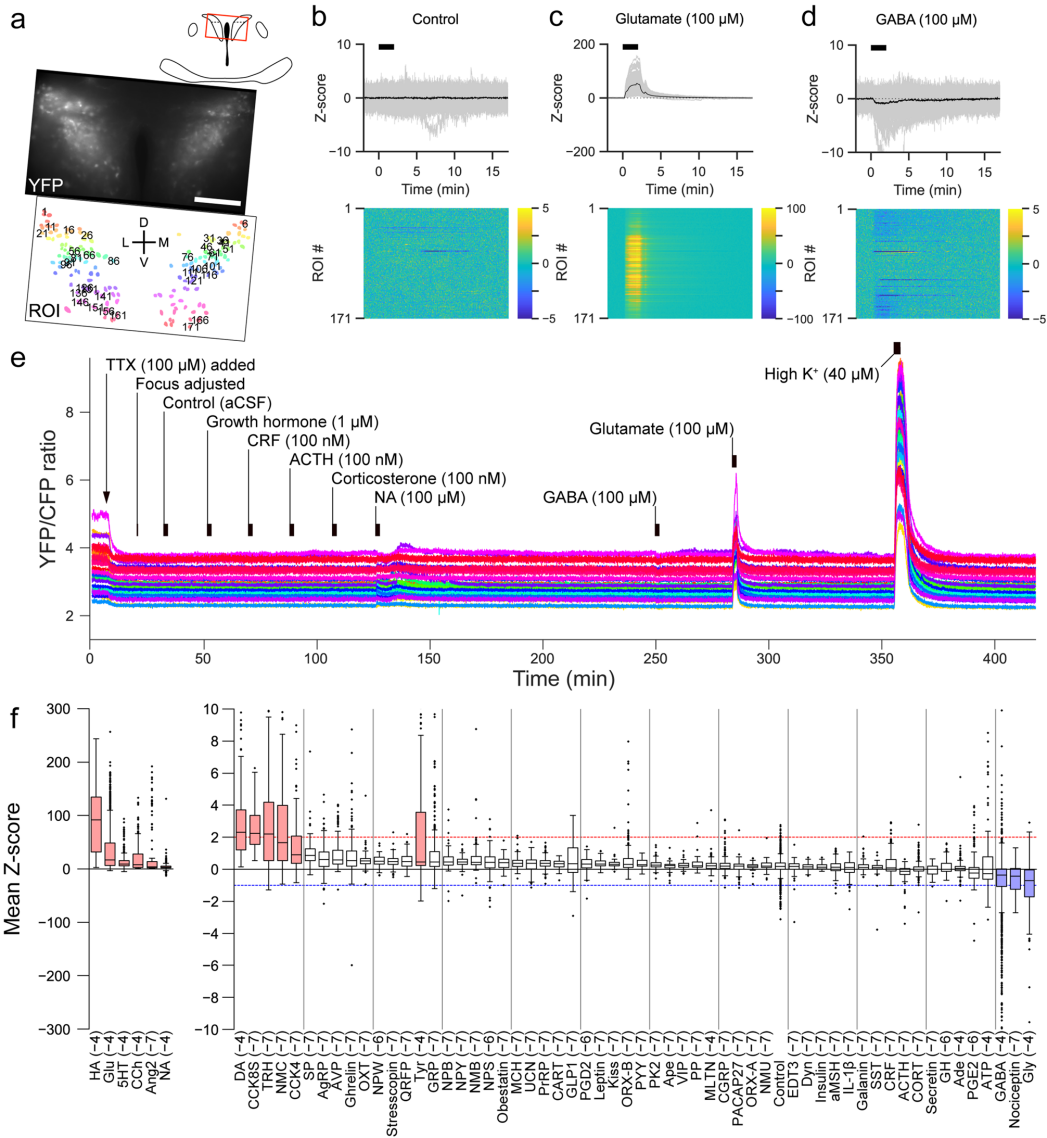
Screen for substances affecting intracellular calcium concentration in PVN-CRF neurons. (**a**) YFP signal at baseline (upper) and in the regions of interest (ROIs, lower) of a representative brain slice. Numbers superimposed over the ROIs align with the dorsoventral axis (from dorsal to ventral). *D* dorsal, *V* ventral, *L* lateral, *M* medial; scale bar: 100 μm. (**b**–**d**) Z-scores of the YFP/CFP ratio recorded from the brain slices shown in (**a**). Upper graphs show traces of individual ROIs (gray) and mean values (black). Black bars indicate the application timing (2 min) of each substance indicated above the graph (**b**, control; **c**, glutamate; **d**, GABA). Heat maps show the Z-scores of individual ROIs indicated by the color bars at right. (**e**) Entire sequence of the YFP/CFP ratio for 1 in every 5 ROIs (35 total ROIs, shown as numbers in **a**) of the brain shown in (**a**)–(**d**). (**f**) Box plots of the calcium signal changes induced by the substances indicated below. In the box plots, the top and bottom of each box indicate the 75% and 25% points, respectively. The line inside the box indicates the median value. Upper and lower ends of the whiskers indicate the points no more than the IQR (interquartile range) × 1.5 from the edge of the box, where IQR = (the value of the 75% point) – (the value of the 25% point). Dots indicate outliers which are data points beyond the whiskers. Abbreviations are listed in Table 1. The compounds are listed in the order of median value. Values in parentheses indicate the Log10 concentration of the substances in mol/L. Red and blue lines indicate where the mean Z-score = 2 and − 1, respectively. Boxes filled in red or blue indicate the substances which increased or decreased intracellular calcium concentrations, respectively.

**Figure 3:**
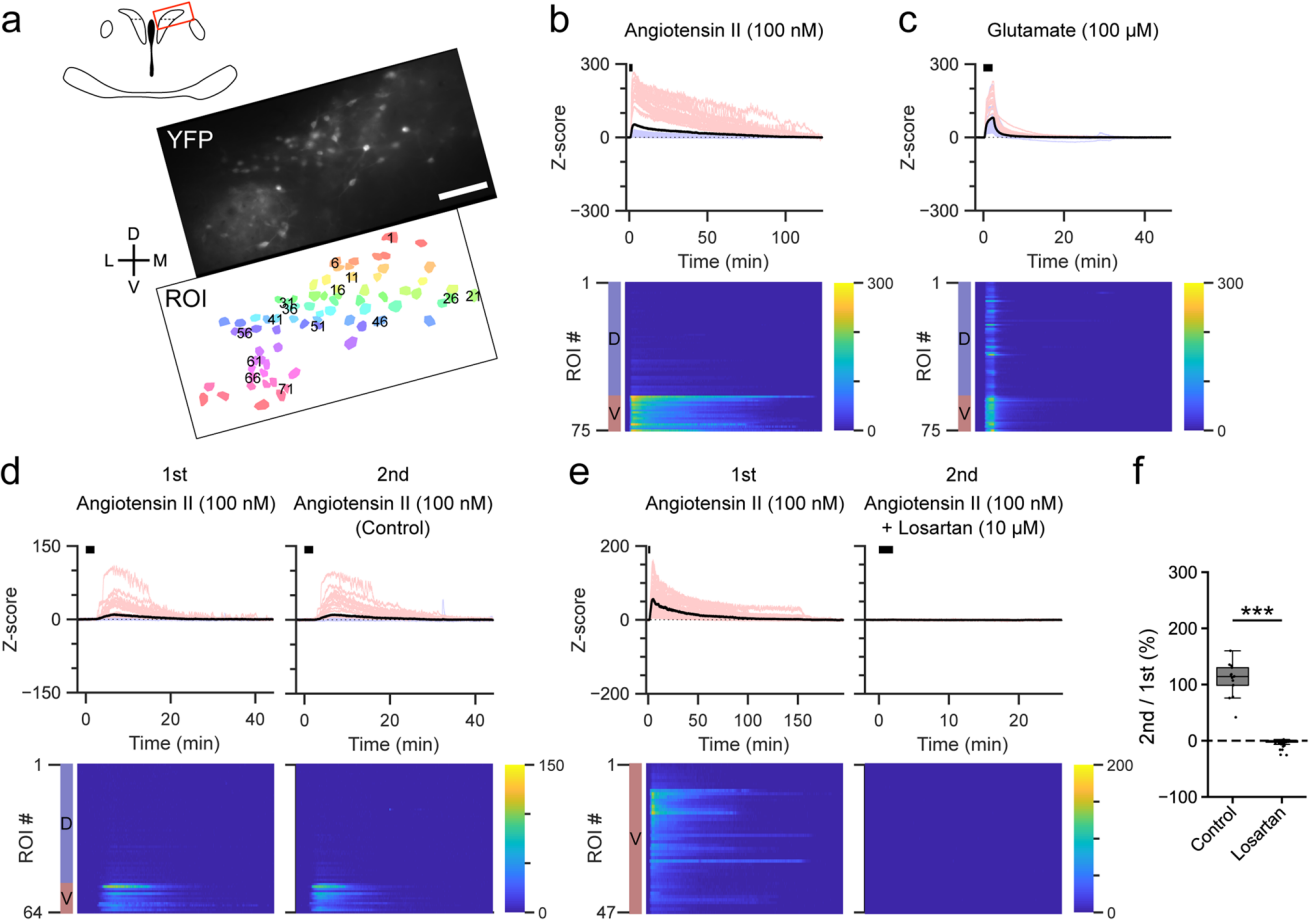
Effect of angiotensin II on PVN-CRF neurons. (**a**) YFP signal (upper) and regions of interest (ROIs, lower) of a representative brain slice. The brain region is indicated by the red box in the upper illustration. Numbers superimposed over the ROIs align with the dorsoventral axis (from dorsal to ventral). *D* dorsal, *V* ventral, *L* lateral, *M* medial; scale bar: 100 μm. (**b**–**e**) Z-scores of the YFP/CFP ratio recorded from each representative brain slice, (**b**) and (**c**) are from the brain slice shown in (**a**). Upper graphs show traces of individual ROIs (light blue: dorsal PVN, light red: ventral PVN) and mean value (black). Black bars indicate the application timing (2 min) of each substance, angiotensin II (AngII) in (**b**), (**d**) and (**e**), glutamate in (**c**). Heat maps show the Z-scores of individual ROIs indicated by the color bars at right. Blue and red color bars at left indicate the dorsal (D) and ventral (V) PVN. AngII was applied twice in (**d**) and (**e**). The type-1 AngII receptor-selective antagonist losartan was applied 5 min before the second application of AngII in (**e**). (**f**) Box plots of ratio of the area under the curve of the Z-scores for the ventral PVN between the first and second application of AngII with and without antagonist. Dots show individual data. ***p < 0.001, Mann–Whitney *U* test.

**Figure 4:**
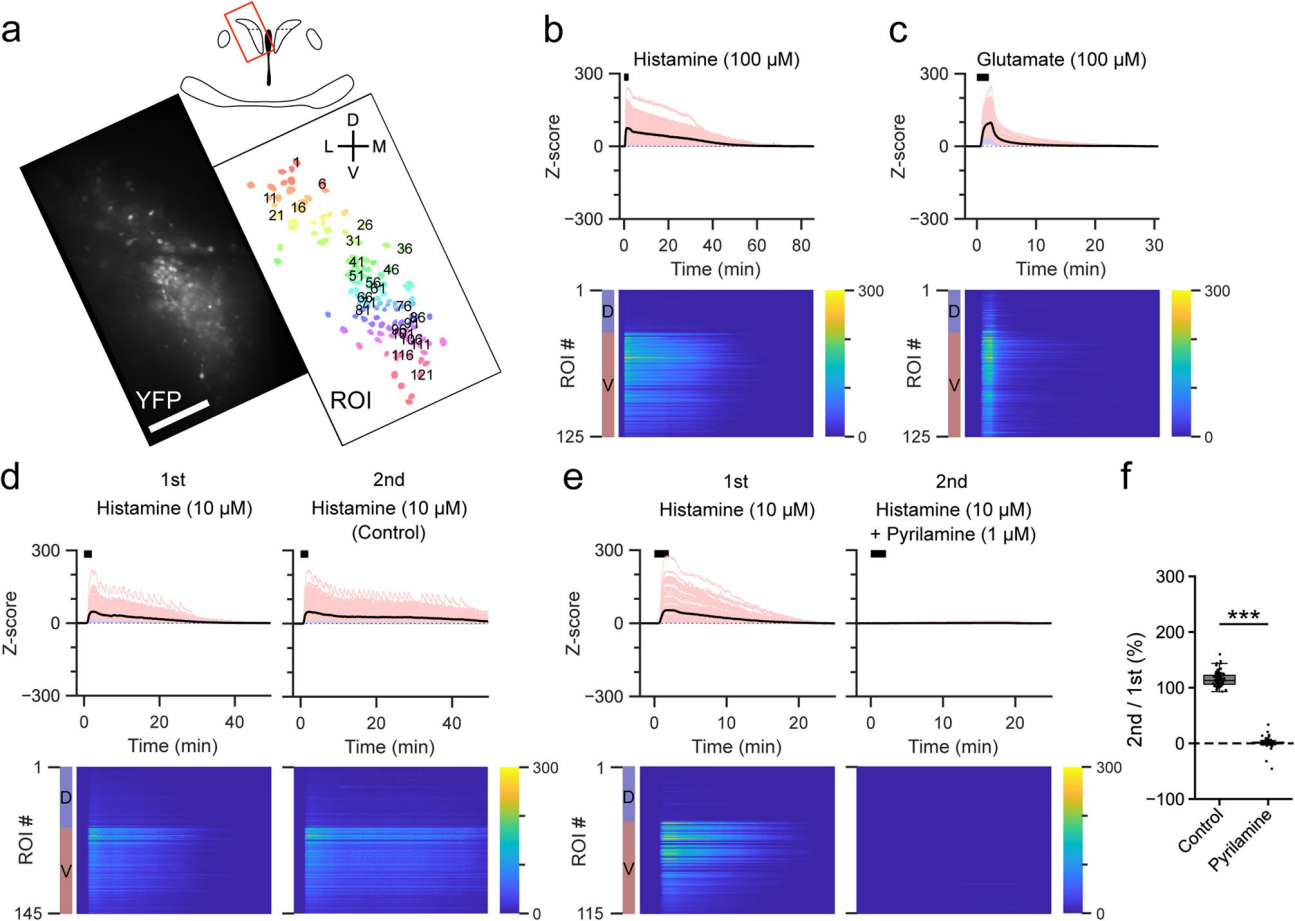
Effect of histamine on PVN-CRF neurons. (**a**) YFP signal (left) and regions of interest (ROIs, right) of a representative brain slice. The brain region is indicated by the red box in the upper illustration. Numbers superimposed over the ROIs align with the dorsoventral axis (from dorsal to ventral). *D* dorsal, *V* ventral, *L* lateral, *M* medial; scale bar: 100 μm. (**b**–**e**) Z-scores of the YFP/CFP ratio recorded from each representative brain slice, (**b**) and (**c**) are from the brain slice shown in (**a**). Upper graphs show traces of individual ROIs (light blue: dorsal PVN, light red: ventral PVN) and mean value (black). Black bars indicate the application timing (2 min) of each substance, histamine (HA) in (**b**), (**d**) and (**e**), glutamate in (**c**). Heat maps show the Z-scores of individual ROIs indicated by the color bars at right. Blue and red color bars at left indicate the dorsal (D) and ventral (V) portions of the PVN. HA was applied twice in (**d**) and (**e**). The histamine H1 receptor-selective antagonist pyrilamine was applied 10 min before the second application of HA in (**e**). (f) Box plots of ratio of the area under the curve of the Z-scores of the ventral PVN between the first and second application of histamine with and without antagonist. Dots show individual data. ***p < 0.001, Mann–Whitney *U* test.

**Figure 5:**
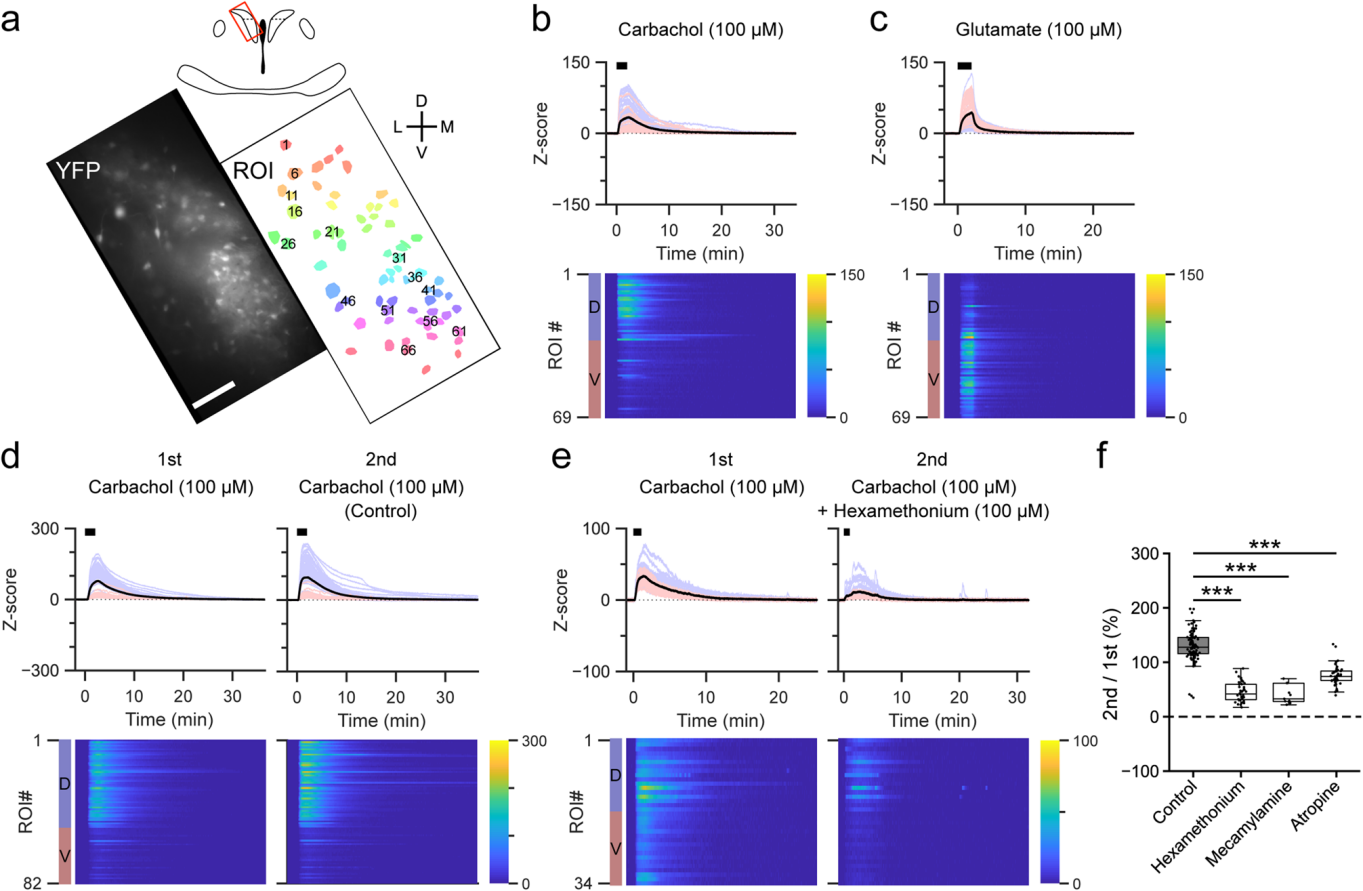
Effect of carbachol on PVN-CRF neurons. (**a**) YFP signal (left) and regions of interest (ROIs, right) of a representative brain slice. The brain region is indicated by the red box in the upper illustration. Numbers superimposed over the ROIs align with the dorsoventral axis (from dorsal to ventral). *D* dorsal, *V* ventral, *L* lateral, *M* medial, scale bar: 100 μm. (**b**–**e**) Z-scores of the YFP/CFP ratio recorded from each representative brain slice, (**b**) and (**c**) are from the brain slice shown in (**a**). Upper graphs show traces of individual ROIs (light blue: dorsal PVN, light red: ventral PVN) and mean value (black). Black bars indicate the application timing (2 min) of each substance, carbachol (CCh) in (**b**), (**d**) and (**e**), glutamate in (**c**). Lower heat maps show Z-scores of individual ROIs indicated by the color bars at right. The blue and red color bars at left indicate the dorsal (D) and ventral (V) portions of the PVN. CCh was applied twice in (**d**) and (**e**). The nicotinic acetylcholine (ACh) receptor-selective antagonist hexamethonium was applied 3 min before the second application of CCh in (**e**). (**f**) Box plots of ratio of the area under the curve of the Z-scores of the dorsal PVN between the first and second application of CCh with and without antagonists: hexamethonium (100 μM), the nicotinic ACh receptor-selective antagonist mecamylamine (1 μM) and the muscarinic ACh receptor selective-antagonist atropine (100 nM). Dots show individual data. ***p < 0.001, Kruskal–Wallis test followed by Dunn’s test.

Next, we performed a screen of bioactive substances to identify those which may affect the activity of PVN-CRF neurons. We prepared acute brain slices from mice expressing YC in their PVN-CRF neurons. Brain slices were mounted in a chamber on a fluorescence microscope and were perfused with artificial cerebrospinal fluid (aCSF). To suppress the indirect effects from synaptic inputs of other neurons, the voltage-gated sodium channel blocker, tetrodotoxin (TTX, 1 μM), was added to the perfused aCSF. We applied candidate substances one by one to the perfused aCSF for 2 min and monitored Y/C ratios during and after application of each substance. We confirmed that aCSF application itself did not affect the Y/C ratio, while the excitatory neurotransmitter glutamate (100 μM) increased the Y/C ratio and the inhibitory neurotransmitter GABA (100 μM) decreased the ratio (Fig. 2a–e). We then screened 63 bioactive substances, including 6 amines, 3 amino acids, 1 choline, 2 lipids, 2 nucleic acids, and 49 peptides (n = 1935 total cells from 26 animals; Table 1, Supplementary Tables S1 and S3). We defined substances which increased or decreased the Y/C ratio from the Z-score, as described in the methods section, to conservatively detect clear calcium changes based on the mean Z-scores observed after application of glutamate and GABA. Results showed that the Y/C ratio was increased by 12 substances: histamine (HA), glutamate, serotonin, carbachol (CCh), angiotensin II (AngII), noradrenaline (NA), dopamine, sulfated cholecystokinin octapeptide (CCK8S), thyrotropin releasing hormone, neuromedin C, cholecystokinin tetrapeptide (CCK4) and tyramine. Conversely, the Y/C ratio was decreased by 3 substances: GABA, nociceptin and glycine (Fig. 2f). Representative calcium signals of the responsive substances are shown in Supplementary Fig. S1, except for glutamate, GABA, HA, CCh and AngII, which are shown in Figs. 2c,d, 4b, 5b and 3b, respectively. Among the responsive substances, CCK8S, CCK4 and tyramine had not been reported in previous studies. We also identified 3 substances, AngII, HA and CCh, that showed different types of calcium responses between the dPVN and vPVN. Therefore, we further examined the effect of these 3 substances using selective antagonists.

**Table 1:** Screened substances.

Substance	Abbreviation	Type	Substance	Abbreviation	Type
Adenosine	Ade	Nucleic acid	Neuromedin B	NMB	Peptide
Adenosine triphosphate	ATP	Nucleic acid	Neuromedin C	NMC	Peptide
Adrenocorticotropic hormone	ACTH	Peptide	Neuromedin U	NMU	Peptide
Agouti-related peptide	AgRP	Peptide	Neuropeptide B	NPB	Peptide
Alpha-melanocyte-stimulating hormone	aMSH	Peptide	Neuropeptide S	NPS	Peptide
Angiotensin II	Ang2	Peptide	Neuropeptide W	NPW	Peptide
Apelin	Ape	Peptide	Neuropeptide Y	NPY	Peptide
Arginine vasopressin	AVP	Peptide	Nociceptin	Nociceptin	Peptide
Carbachol	CCh	Choline	Noradrenaline	NA	Amine
Carcitonin gene-related peptide	CGRP	Peptide	Obestatin	Obestatin	Peptide
Cholecystokinin tetrapeptide	CCK4	Peptide	Orexin-A	ORX-A	Peptide
Cocaine- and amphetamine-related transcript	CART	Peptide	Orexin-B	ORX-B	Peptide
Corticotropin releasing factor	CRF	Peptide	Oxytocin	OXT	Peptide
Cortisol	CORT	Steroid	Pancreatic polypeptide	PP	Peptide
Dopamine	DA	Amine	Peptide YY	PYY	Peptide
Dynorphin A	Dyn	Peptide	Pituitary adenylate cyclase-activating polypeptide	PACAP27	Peptide
Endothelin-3	EDT3	Peptide	Prokineticin-2	PK2	Peptide
Galanin	Galanin	Peptide	Prolactin-releasing peptide	PrRP	Peptide
Gamma-aminobutyric acid	GABA	Amino acid	Prostaglandin D2	PGD2	Lipid
Gastrin-releasing peptide	GRP	Peptide	Prostaglandin E2	PGE2	Lipid
Ghrelin	Ghrelin	Peptide	Pyroglutamylated RF amide peptide	QRFP	Peptide
Glucagon-like peptide-1	GLP1	Peptide	Secretin	Secretin	Peptide
Glutamate	Glu	Amino acid	Serotonin (5-hydroxytriptamine)	5HT	Amine
Glycine	Gly	Amino acid	Somatostatin	SST	Peptide
Growth hormone	GH	Peptide	Stresscopin	Stresscopin	Peptide
Histamine	HA	Amine	Substance P	SP	Peptide
Insulin	Insulin	Peptide	Sulfated cholecystokinin octapeptide	CCK8S	Peptide
Interleukin-1β	IL-1β	Peptide	Thyrotropin-releasing hormone	TRH	Peptide
Kisspeptin	Kiss	Peptide	Tyramine	Tyr	Amine
Leptin	Leptin	Peptide	Urocortin-3	UCN	Peptide
Melanin-concentrating hormone	MCH	Peptide	Vasoactive intestinal peptide	VIP	Peptide
Melatonin	MLTN	Amine			

Substances used for screening are shown in the table listed by substance (1st column), abbreviation (2nd column) and type (3rd column).

### Angiotensin II increased [Ca^2+^]_i_ in the ventral portion of PVN-CRF neurons

AngII is a bioactive peptide known to be involved in the regulation of fluid homeostasis^25^. AngII is also reported to be produced in the brain from angiotensinogen via angiotensin I by successive digestion by renin and angiotensin converting enzyme^26^. AngII functions to induce water intake in the central nervous system^27^. Application of AngII (100 nM) induced a large and long-lasting increase in the Y/C ratio in PVN-CRF neurons. This response was larger in vPVN-CRF neurons compared to dPVN-CRF neurons (Fig. 3a,b). The glutamate-induced increase in the Y/C ratio in both the vPVN and dPVN of the same slice preparation confirmed that neurons from both portions were healthy (Fig. 3c). To identify the receptors implicated in the AngII-induced [Ca^2+^]_i_ increase, we perfused a type-1 AngII (AT_1_) receptor-selective antagonist, losartan (10 μM), for 10 min prior to AngII application. This resulted in complete inhibition of the AngII-induced increase in the Y/C ratio (Fig. 3d–f, Supplementary Fig. S2a, Supplementary Tables S2 and S4). Antagonist application alone did not cause a clear change in the Y/C ratio (Supplementary Fig. S3). This is consistent with a previous report that PVN-CRF neurons express type-1a AngII (AT_1a_) receptors^28^, and that the AT_1a_ receptor is known to couple with the Gq family of G proteins^29^. Thus, our results suggest that AngII increases [Ca^2+^]_i_ in vPVN-CRF neurons via the AT_1_ receptor.

### Histamine increases [Ca^2+^]_i_ in the ventral portion of PVN-CRF neurons

HA is a monoamine released from histaminergic neurons as a neurotransmitter in the brain^30^. Histaminergic neurons are known to be involved in various physiological functions^31^, such as the maintenance of wakefulness^32^. Application of HA (100 μM) induced a transient [Ca^2+^]_i_ increase in PVN-CRF neurons. This increase was larger in vPVN-CRF neurons compared with dPVN-CRF neurons (Fig. 4a,b), in contrast to glutamate which induced an increase in the Y/C ratio in both the dPVN and vPVN within the same slice preparation (Fig. 4c). To identify receptors that may play a role in the [Ca^2+^]_i_ increase in vPVN-CRF neurons, a histamine H_1_ (H_1_) receptor-selective antagonist, pyrilamine (1 μM), was perfused for 10 min prior to HA application. This resulted in the complete inhibition of the HA-induced increase in the Y/C ratio by co-application of pyrilamine (Fig. 4d–f, Supplementary Fig. S2b, Supplementary Tables 2 and 4). Antagonist application alone did not result in a clear change in the Y/C ratio (Supplementary Fig. S3). It has been reported that central administration of HA, histamine H_1_ and H_2_ receptor selective agonists increases *Crh* mRNA in the PVN-CRF neurons^33^. This increase in mRNA expression might reflect activation of PVN-CRF neurons by HA since the H_1_ receptor is known to couple with the Gq family of G proteins^34^. Thus, our results suggest that HA increases [Ca^2+^]_i_ in vPVN-CRF neurons via the H_1_ receptor.

### Carbachol increases [Ca^2+^]_i_ in the dorsal portion of PVN-CRF neurons

CCh is an agonist of both nicotinic and muscarinic acetylcholine receptors (nACh and mACh, respectively). Acetylcholine (ACh) is derived from choline and produced in cholinergic neurons, and is known to be involved in various physiological functions^35^ such as memory, learning and wakefulness^36^. Application of CCh (100 μM) induced a larger increase in the Y/C ratio in the dPVN than vPVN (Fig. 5a,b), while glutamate induced an increase in the Y/C ratio in both the dPVN and vPVN within the same slice preparation (Fig. 5c). Since this increase in the Y/C ratio can be induced via nACh receptors and/or mACh receptors, we tried to discriminate between the receptors implicated in the [Ca^2+^]_i_ increase. First, we perfused the nACh receptor-selective antagonist, hexamethonium (100 μM), for 3 min prior to CCh application. This resulted in a partially diminished CCh-induced increase in the Y/C ratio (45.4 ± 2.8% compared with CCh only, n = 98 cells; Fig. 5d–f, Supplementary Fig. S2c, Supplementary Tables 2 and 4). To further confirm the involvement of the nACh receptor in this response, another nACh receptor-selective antagonist, mecamylamine (1 μM), was used. Similar to hexamethonium, mecamylamine partially inhibited the [Ca^2+^]_i_ increase (40.1 ± 5.1%, n = 11 cells; Fig. 5f, Supplementary Fig. S2c, Supplementary Tables 2 and 4). Next, to determine the involvement of the mACh receptor, a mACh receptor-selective antagonist, atropine (100 nM), was tested. Atropine partially diminished the CCh-induced increase in the Y/C ratio (75.1 ± 2.6%, n = 48 cells; Fig. 5f, Supplementary Fig. S2c, Supplementary Tables 2 and 4). Antagonist application alone did not result in a clear change in the Y/C ratio (Supplementary Fig. S3). It has been reported that microinjection of ACh increases *Crh* mRNA expression in the PVN^37^. It is possible that this increase in mRNA expression could be explained by the direct effect of ACh on PVN-CRF neurons. Thus, our results suggest that CCh increases [Ca^2+^]_i_ in the dPVN via both nACh receptors and mACh receptors.

## Discussion

**Figure 6:**
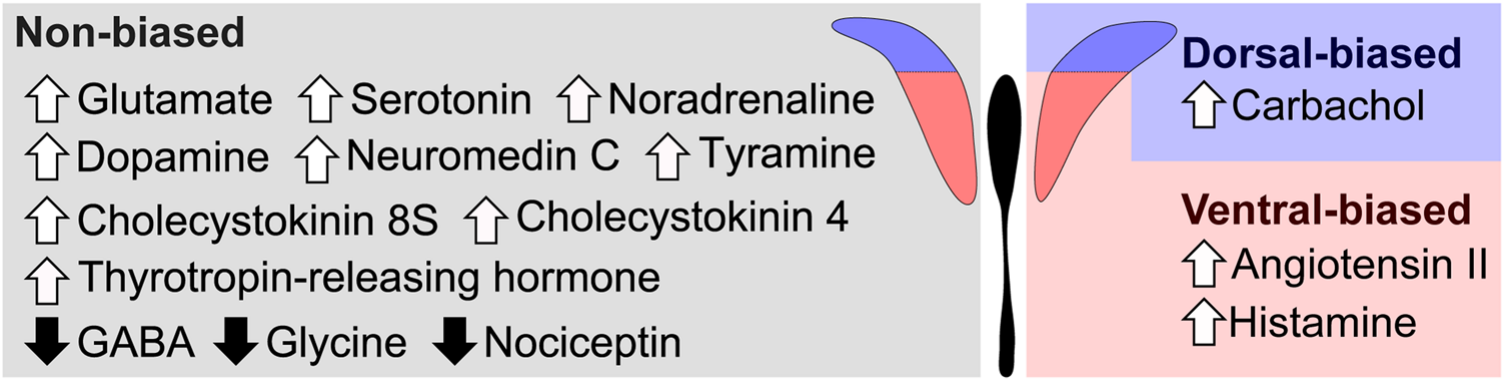
Substances affecting intracellular calcium concentrations in PVN-CRF neurons. Summary of substances which increased or decreased (white upward or black downward arrows shown to the left of each substance, respectively) intracellular calcium concentration in PVN-CRF neurons. Substances listed in the left panel affected both dorsal- and ventral-CRF neurons (non-biased). In contrast, carbachol seemed to mainly affect the dorsal-CRF neurons (dorsal-biased), whereas angiotensin II and histamine mainly affected the ventral-CRF neurons (ventral-biased).

In this study, we found 15 candidate physiological substances affecting activity of PVN-CRF neurons (Fig. 6). These substances are possibly implicated in various physiological functions through activation or inhibition of PVN-CRF neurons. For example, activity of PVN-CRF neurons is increased or decreased by negative or positive valence, respectively^2^. Specifically, optogenetic activation or inhibition of PVN-CRF neurons induced or reduced stress-related behavior, respectively^2, 38^. This kind of behavior-inducible neuronal activity can be mediated by biophysiological substances. For example, AngII is thought to be involved in the stress response via the PVN^39^. The expression of AT_1_ receptors is reported to be increased in the PVN following stress^40^. Furthermore, the AT_1a_ receptor in the PVN was shown to be essential for the expression of anxiety behavior^41^. However, these studies did not reveal the direct involvement of PVN-CRF neurons in these responses since they did not directly manipulate PVN-CRF neurons. A recent study identified that the AT_1a_ receptor is exclusively expressed in PVN-CRF neurons, and showed that AngII is implicated in the stress response via PVN-CRF neurons^28^.

Although our screening system identified many novel substances which affect the activity of PVN-CRF neurons, it might not have detected all substances that can affect PVN-CRF neurons. For example, glucagon-like peptide-1 (GLP-1) expressing neurons are reported to innervate PVN-CRF neurons, as observed by electron microscopy^42^. This effect on PVN-CRF neurons was confirmed by electrophysiology combined with optogenetics^43^. GLP-1 was shown to directly increase [Ca^2+^]_i_ in isolated PVN-CRF neurons^44^. In our screening system, a small number of PVN-CRF neurons showed an increase in [Ca^2+^]_i_ in response to GLP-1; however, we classified GLP-1 as “not-responsive” since most of the neurons did not show a sufficient response (Fig. 1f). This difference can be attributed to the fact that we used simple criteria to define “responsive” substances; we used just a single threshold and the majority of the neural population to determine if there was an “increasing” or “decreasing” effect on [Ca^2+^]_i_. Although a previous study showed that the Y_1_ receptor^45^ and EP_4_ receptor^46^ are expressed in PVN-CRF neurons, our screening system did not show clear changes in [Ca^2+^]_i_ by application of ligands for these receptors, i.e. neuropeptide Y and prostaglandin E_2_. These results suggest that substances classified as “not-responsive” in our screen might represent false negatives since [Ca^2+^]_i_ is just one of the indicators of neural activity.

Furthermore, this study did not address the more detailed mechanisms that regulate PVN-CRF neuronal activity. For example, in our experiments we added TTX to suppress multi-synaptic modulation^47^ of PVN-CRF neurons. Therefore, it is possible that substances classified as not-responsive might affect the activity of PVN-CRF neurons in an indirect manner via their effects on other neurons. The possible existence of TTX-resistant sodium channels (Na_v_1.5, Na_v_1.8 and Na_v_1.9)^48^ might also be taken into consideration. Among these channels, Na_v_1.8 is reported to be expressed in the PVN; however, its sodium channel current (*I*_*Na*_) accounts for less than 2% of the total *I*_*Na*_^49^. In addition, we sequentially screened multiple substances; however, GPCR itself can be modulated by specific substances via some mechanisms, such as desensitization/resensitization^50^ and heterodimerization^51, 52^. As shown in Supplementary Table S3, we applied each substance in different orders in multiple brain slices from different animals to minimize potential effects from the precedingly applied substances. Recently, sexual dimorphism has been reported in the function of an NMDA receptor subunit in CRF-producing neurons^53, 54^. It is thus possible that this type of sexual dimorphism could exist for other receptors. Since we randomly used both male and female animals in this study, we could not examine potential sexual dimorphism. However, the methods we adopted in the present study are flexible and can also be applied to evaluating the complex regulatory mechanisms of PVN-CRF activity by selecting the appropriate conditions such as the absence of TTX, presence of other antagonists, combinations of substances or evaluation in either sex.

Our screening system showed that dPVN-CRF neurons and vPVN-CRF neurons have different features in their response to various substances. For example, AngII and HA increased [Ca^2+^]_i_ mainly in the vPVN-CRF neurons, which makes a contrast to the observation that CCh increased [Ca^2+^]_i_ mainly in the dPVN-CRF neurons. The major proportion of the vPVN-CRF neurons have been well-recognized to project to the median eminence and regulate the HPA-axis, whereas part of the dPVN-CRF neurons are known to project to pre-autonomic neurons in the lower brain stem, and possibly to the spinal cord, and they may be involved in the regulation of autonomic output from the spinal preganglionic neurons^11^. Therefore, we assume that the differences in responses between the vPVN-CRF neurons and dPVN-CRF neurons could be related to the differences in expression of respective receptors among CRF neuronal subclasses, which are involved in different physiological functions. It was reported that α7 subunit-containing nicotinic acetylcholine (α7nACh) receptors are expressed in magnocellular neurons and not in parvocellular neurons^55^, but it is not clear whether α7nACh receptors are expressed in a particular subclass of CRF neurons in the dPVN, which needs to be examined further. On the other hand, AngII induced larger increases in [Ca^2+^]_i_ in some vPVN-CRF neurons compared to the responses in other neurons in the same anatomical location (Fig. 3b). This makes a contrast to the more uniform [Ca^2+^]_i_ increase when it was induced by glutamate (Fig. 3c). These results imply the potential existence of subpopulations among the vPVN-CRF neurons. It is necessary to confirm distribution of receptor expression more in detail to understand the mechanism underlying the present results.

It is not clear whether the changes in calcium signals, observed in the present study, are the consequences of electrophysiological firing of the PVN-CRF neurons. Calcium signals can be affected not only by voltage-gated calcium channels sensitive to changes in membrane potential, but also by additional machinery, including other calcium pumps and transporters, as well as the transfer of Ca^2+^ among cytosol and other organelles such as in mitochondria and the endoplasmic reticulum^56^. For example, NA is reported to increase the levels of phospho-extracellular signal-regulated kinases 1/2 (ERK1/2) in PVN-CRF neurons via the α_1_ adrenoreceptor^57^, while ERK1/2 can be regulated by intracellular calcium signaling to induce changes in gene expression^58^. It is also reported that NA activates phospholipase C via the α_1_ adrenoreceptor, and can subsequently induce an increase in [Ca^2+^]_i_ through both the release of intracellular Ca^2+^ stores as well as extracellular Ca^2+^ influx in isolated PVN neurons^59^. It is possible that the changes in [Ca^2+^]_i_ observed in the present study might be implicated in such intracellular calcium signaling, independent of electrophysiological changes.

NA is known to act as an important regulator of PVN-CRF neurons^60^. However, the mechanism by which it regulates PVN-CRF neuronal activity may not be uniform, and the presence of multiple underlying mechanisms has been proposed. For example, low concentrations of NA increased firing of neurons in the PVN, whereas high concentrations of NA decreased firing of them^61^. NA has also been shown to indirectly increase the frequency and amplitude of excitatory post-synaptic current in parvocellular neurons in the PVN via glutamatergic interneurons expressing α_1_ adrenoreceptors; while, conversely, NA can also directly hyperpolarize parvocellular neurons via the β adrenoreceptors^62^. Furthermore, NA is known to increase [Ca^2+^]_i_ in isolated PVN neurons via the α_1_ receptors^59^. In our study, although the mean Z-score of the NA-induced [Ca^2+^]_i_ increase was smaller than that of the other 5 substances (Fig. 2f), the [Ca^2+^]_i_ signal transiently dropped in the middle of an increment, also suggesting the presence of complex regulatory mechanism (Supplementary Fig. S1b). More detailed experiments are required to examine the effect of NA on PVN-CRF neurons.

In summary, our method for calcium imaging provided a precise and reliable way to compare the effect of various substances on the activity of PVN-CRF neurons. The present study will contribute to the understanding of the mechanisms of stress responses, and to the development of future studies aimed at elucidating the actual physiological function of the calcium-modulating effects of the substances identified in this screen.

## Methods

### Animals

All experiments were carried out in accordance with Nagoya University Regulations on Animal Care and Use in Research. All experiments were approved by the Institutional Animal Care and Use Committees of the Research Institute of Environmental Medicine, Nagoya University, Japan (approval #19232 and #19268). All efforts were made to reduce the number of animals used and to minimize the pain and suffering of animals. Adult (> 4 weeks old) *CRF-iCre* (*Crh*^*tm2*(*icre*)*Ksak*^) mice^21^ and mice generated by crossing with *Ai14* (B6;129S6-*Gt*(*ROSA*)*26Sor*^*tm14*(*CAG-tdTomat*o*)Hze*^*/J*) mice were used in this study. Animals were maintained on a 12-h light–dark cycle under ad libitum feeding and drinking conditions. Room temperature was maintained at 23 ± 2 °C.

### Buffers

The following buffers were used in this study: phosphate buffered saline (PBS) containing (in mM) 137 NaCl, 2.7 KCl, 8 Na_2_HPO_4_ and 1.5 KH_2_PO_4_; KCl-based pipette solution containing 145 KCl, 1 MgCl_2_, 10 HEPES, 1.1 EGTA, 2 adenosine-5′-triphosphate magnesium salt and 0.5 guanosine-5′-triphosphate disodium salt, 280–290 mOsm, pH 7.3 with KOH; cutting solution containing 15 KCl, 3.3 MgCl_2_, 110 K-gluconate, 0.05 EGTA, 5 HEPES, 25 glucose, 26.2 NaHCO_3_ and 0.0015 ± -3-(2-carboxypiperazin-4-yl)propyl-1-phosphonic acid; and aCSF containing 124 NaCl, 3 KCl, 2 MgCl_2_, 2 CaCl_2_, 1.23 NaH_2_PO_4_, 26 NaHCO_3_, 25 glucose. Cutting solution and aCSF were bubbled with mixed gas (O_2_, 95%; CO_2_, 5%).

### Plasmids

The *pAAV-CMV-FLEX-YC-Nano50-WPRE* plasmid was produced in-house with a *Yellow Camelon-Nano50/pcDNA3* plasmid (Addgene #51964). The *pHelper* plasmid was purchased from Agilent Technologies and *pAAV-RC* (serotype 9) was kindly provided by the University of Pennsylvania vector core.

### Adeno-associated virus (AAV)

AAV9-CMV-FLEX-YC-Nano50-WPRE (1.0 × 10^13^ copies/mL) was generated according to the protocol described in the supplementary methods. Briefly, *pHelper*, *pAAV-RC* (serotype 9) and *pAAV-CMV-FLEX-YC-Nano50-WPRE* plasmids were transfected into AAV-293 cells (Agilent Technologies) using the calcium phosphate method. Three days after transfection, cells were collected and suspended in PBS and AAVs were purified by ultracentrifugation. The final virus solvent was a mixture of PBS and OptiPrep (Alere Technologies AS), where the ratio of the solution depended on the AAV titer.

### Virus vector injection

Mice were anesthetized with 1–2% isoflurane and fixed on a stereotaxic frame. The scalps were opened and the skull above the injection site was drilled. A glass pipette (GC150-10; Harvard Apparatus) made with a puller (P-97, Sutter Instrument) was used for injection of AAV. In the bilateral PVN (in mm, AP − 0.5 from the bregma, ML 0.5 from the midline, DV − 4.2 from the brain surface), 600 μL/site of AAV solution was injected by air pressure pulses regulated by a Pneumatic Picopump (World Precision Instruments) with a pulse generator (SEN-7103, Nihon Kohden). The injected animals were used for subsequent experiments at least 3 weeks after injection.

### Fixed brain slices

Mice were deeply anesthetized with isoflurane and perfused with 25 mL chilled saline followed by 25 mL chilled 10% formalin. After decapitation, each skull was carefully removed and brains were placed into chilled 10% formalin for post-fixation overnight. After post-fixation, brains were placed into 30% sucrose containing PBS for cryoprotection for at least 48 h. After cryoprotection, brains were placed into O.C.T. compound (Sakura Finetek Japan) and frozen at − 80 °C for 20 min then placed into a − 20 °C cryostat (CM3050 S; Leica Biosystems). Embedded brains were fixed on a stage using O.C.T. compound and sliced at a thickness of 40 μm.

### Cell counts

Every 1 in 4 cryo-sectioned brain slices were used for cell counts. Slices were mounted on slide glasses and were visualized and imaged using a confocal microscope (LSM710, Carl Zeiss). Images of YFP and tdTomato fluorescence were obtained and the number of cells positive for each signal was counted manually.

### Acute brain slices

Animals were anesthetized with isoflurane and decapitated. The brains were immediately removed from the head and incubated in ice-cold cutting solution. Brains were sliced at a thickness of 250 μm using a vibratome (VT1200S, Leica). The slices were incubated in aCSF at 35ºC for 1 h then at room temperature for at least 1 h.

### Substances for screening

The compounds used for screening are listed in Table 1.

### Calcium imaging

Brain slices were placed in a chamber perfused with aCSF at 1.5 mL/min. Slices were anchored with a harp to avoid movement. A microscope (BX51WI, Olympus) was equipped with two objective lenses (20 × and 40 ×), a filter cube with a dichroic mirror for CFP excitation, an optical splitter (W-VIEW GEMINI, Hamamatsu photonics) with band-pass emitters and a dichroic mirror for YFP/CFP recording, an electron-multiplying charge-coupled device (EMCCD) camera (iXon Ultra 897 or iXon Ultra 888, Andor, Oxford Instruments) and a light source (Spectra X, Lumencor). For excitation, blue light (440 ± 20 nm, 50–210 μW/mm^2^, 100 ms) was applied. The fluorescent signals for CFP and YFP were observed and recorded using software (MetaFluor, Molecular Devices).

### Electrophysiological recordings

A glass pipette was made from a glass capillary (GC150-10, Harvard Apparatus) using a puller (P-1000, Sutter Instrument) to have a pipette resistance of 4–10 MΩ. KCl-based pipette solution was used as the internal solution. For patch-clamp recordings, an amplifier (Axopatch 200B, Molecular Devices) and a digitizer (Axon Digidata 1550A, Molecular Devices) were used. After identifying a cell expressing YC, the cell was contacted and ruptured with a glass pipette and maintained in a whole-cell current clamp mode. Negative current was injected to suppress spontaneous firing. Once the resting membrane potential was stable for > 30 s, command current (300 pA, 5 ms) was injected with a specific frequency (1, 2, 5 and 10 Hz) for 10 s sequentially with a gap of more than 1 min between each frequency. Data was acquired using software (Clampex 10.7, Molecular Devices).

### Substance screening

To monitor cell autonomous effects and to suppress the effects of synaptic inputs from other neurons, the voltage-gated sodium channel blocker tetrodotoxin (1 μM) was added to the aCSF. For a single brain slice, 10 substances, at most, were screened sequentially. Each candidate substance was dissolved in aCSF. The solution was then applied for 2 min via perfusion. The time between applications was at least 5 min. When any change in calcium signal was observed, the next substance was not applied until the signal returned to baseline and was stable for an additional 5 min. As controls, for detection of baseline, increased and decreased calcium concentrations, aCSF, glutamate and GABA were applied, respectively. Each substance was examined at least twice in different orders in multiple slices from different animals (Supplementary Table S3).

### Antagonist experiments

To examine the effects of an antagonist, the antagonist was applied for at least 3 min before the onset of the subject substance application. The subject substance was diluted into the antagonist solution and applied for 2 min. The antagonist was further applied for at least 5 min after the offset of substance application. After antagonist application, normal aCSF was perfused.

### Analysis

Images of YFP and CFP were motion corrected and aligned if needed using an original program based on the Scale-Invariant Feature Transform^63^ written in MATLAB (R2019a, MathWorks). Regions of interest (ROIs) were drawn to surround cell bodies. ROIs that included two or more cells, or cells that disappeared before the end of an experiment were omitted. The intensity of YFP and CFP was measured using Fiji^64^, and subsequent calculations including the Y/C ratio were performed in MATLAB.

For patch-clamp recordings, the raw Y/C ratio was used for analysis. The peak Y/C ratio during current injection of each stimulation was subtracted by the mean Y/C ratio during the period 30 s before current injection (R_0_) to obtain the peak ΔR. ΔR was divided by R_0_ to obtain the peak ΔR/R_0_.

For screening and antagonist experiments, the value of the Y/C ratio was corrected using the calculations described in the supplementary methods.

For screening, the mean Z-score during the 5 min after the onset of each substance application was used. Quartiles of the mean Z-score for each substance were calculated from the combined screening experiments. When the third quartile was greater than 2, the substance was defined as increasing [Ca^2+^]_i_; conversely, when the first quartile was less than − 1, the substance was defined as decreasing [Ca^2+^]_i_. These definitions were established to conservatively detect a clear calcium change based on mean Z-scores observed after application of glutamate and GABA.

For antagonist experiments, the integral of the Z-score during the 5 min after onset of subject substance application was calculated for both the 1st (substance only) and 2nd (with or without antagonist) sessions (Z_1st_ and Z_2nd_, respectively). Z_2nd_ was divided by Z_1st_ to obtain the 2nd/1st ratio for control and antagonist effects.

### Statistics

All data were presented as the mean ± standard error of the mean (s.e.m.). Statistical analysis was performed using software (OriginPro, Version 2019, OriginLab). A p-value less than 0.05 was considered statistically significant. To test normality, the Shapiro–Wilk test was performed. When data did not show normality, to test differences in distribution between control and subject compounds, the Mann–Whitney *U* test was performed. To test population differences, a Kruskal–Wallis test followed by the Dunn’s test was performed.

## Supplementary information

Supplementary Information.

## Data Availability

The datasets generated during and/or analysed during the current study are available from the corresponding author on reasonable request.
