# Supplementary material for: Identification of substances which regulate activity of corticotropin-releasing factor-producing neurons in the paraventricular nucleus of the hypothalamus
Source: Sci Rep. 2020 Aug 12;10:13639. doi: 10.1038/s41598-020-70481-5 (PMC7424526; doi:10.1038/s41598-020-70481-5)
Supplement: Supplementary file 1 — Supplementary Information. [file 41598_2020_70481_MOESM1_ESM.pdf]

Identification of substances which regulate activity of corticotropin-releasing factor-producing neurons in the paraventricular nucleus of the hypothalamus

Yasutaka Mukai[1,2,3,4], Ayako Nagayama[1,2], Keiichi Itoi[5], Akihiro Yamanaka[1,2,3]*

[1]Department of Neuroscience II, Research Institute of Environmental Medicine, Nagoya University, Nagoya 464-8601, Japan
[2]Department of Neural Regulation, Nagoya University Graduate School of Medicine, Nagoya 466-8550, Japan
[3]CREST, JST, Honcho Kawaguchi, Saitama 332-0012, Japan
[4]JSPS Research Fellowship for Young Scientists, Tokyo 102-0083 Japan
[5]Department of Neuroendocrinology, Graduate School of Medicine, Tohoku University, Sendai 980-8575, Japan

Supplemental Materials and Methods

Animals

All experiments were carried out in accordance with Nagoya University Regulations on Animal Care and Use in Research. All experiments were approved by the Institutional Animal Care and Use Committees of the Research Institute of Environmental Medicine, Nagoya University, Japan (approval #19232 and #19268). All efforts were made to reduce the number of animals used and to minimize the pain and suffering of animals. Adult (>4 weeks old) *CRF-iCre* (*Crh*$^{tm2(icre)Ksak}$) mice[1] and mice generated by crossing with *Ai14* (B6;129S6-*Gt(ROSA)26Sor*$^{tm14(CAG\text{-}tdTomato)Hze}$/*J*) mice were used in this study. Animals were maintained on a 12-hour light-dark cycle under *ad libitum* feeding and drinking conditions. Room temperature was maintained at 23 ± 2˚C.

Buffers

The following buffers were used in this study: 2×HEPES-buffered saline (HBS) containing (in mM) 280 NaCl, 1.5 Na_2HPO_4 and 50 HEPES, and pH adjusted to 7.10 with NaOH; virus dilution buffer (VDB) containing 10 Tris-HCl, 50 KCl and 5 $MgCl_2$; phosphate buffered saline (PBS) containing 137 NaCl, 2.7 KCl, 8 Na_2HPO_4 and 1.5 KH_2PO_4; KCl-based pipette solution containing 145 KCl, 1 $MgCl_2$, 10 HEPES, 1.1 EGTA, 2 adenosine-5'-triphosphate magnesium salt and 0.5 guanosine-5'-triphosphate disodium salt, 280–290 mOsm, pH 7.3 with KOH; cutting solution containing 15 KCl, 3.3 $MgCl_2$, 110 K-Gluconate, 0.05 EGTA, 5 HEPES, 25 Glucose, 26.2 $NaHCO_3$ and 0.0015 ±-3-(2-Carboxypiperazin-4-yl)propyl-1-phosphonic acid; and aCSF containing 124 NaCl, 3 KCl, 2 $MgCl_2$, 2 $CaCl_2$, 1.23 NaH_2PO_4, 26 $NaHCO_3$, 25 glucose. Cutting solution and aCSF were bubbled with mixed gas (O2, 95%; CO2, 5%).

Plasmids

The *pAAV-CMV-FLEX-YC-Nano50-WPRE* plasmid was produced in-house. A YC-Nano50 encoding a DNA fragment between the *Hind*III and *Xba*I sites of the *Yellow Camelon-Nano50/pcDNA3* plasmid (provided by Dr. T. Nagai of Osaka University, identical to Addgene #51964) was inserted into a locus between the *Hind*III and *Xba*I sites of a lab-made *pAAV-CMV-FLEX-MCS-WPRE* plasmid. The *pHelper* plasmid was purchased from Agilent Technologies (Santa Clara, CA, USA) and *pAAV-RC* (serotype 9) was kindly provided by the University of Pennsylvania vector core.

Adeno associated virus (AAV)

AAV9-CMV-FLEX-YC-Nano50-WPRE (1.0×10^{13} copies/mL) was produced in-house. *pHelper*, *pAAV-RC* (serotype 9) and *pAAV-CMV-FLEX-YC-Nano50-WPRE* plasmids (200 µg, each) were mixed into 16 mL 0.3 M $CaCl_2$ in a 50 mL centrifuge tube. The solution was further mixed with 16 mL

2×HBS by vortexing. Eight mL/dish of the calcium-phosphate DNA mixture was transferred into four 25 cm × 25 cm dishes in which AAV-293 cells (Agilent Technologies) were cultured in medium (Dulbecco's Modified Eagle's Medium – high glucose, Sigma-Aldrich, Merck, Darmstadt, Germany). The transfected cells were incubated in a CO_2 incubator at 37˚C. The medium was changed to fresh medium within 6 to 24 hours after transfection. Three days after transfection, cells were collected using a scraper and centrifuged at 1,000 rpm at room temperature (RT) for 10 min. The supernatant was exchanged with PBS and vortexed to become homogenous. The solution was centrifuged at 1,000 rpm at RT for another 10 min. The supernatant was exchanged with 16 mL fresh PBS and suspended again. The cell suspension solution was frozen at −80˚C for 15 min, incubated at 37˚C for 10 min and vortexed at RT for 1 min. This freeze/thaw cycle was repeated 4 times. The cell suspension was then mixed with 6 μL Benzonase Nuclease (Millipore, Merck) and incubated at 37˚C for 1 hour. The solution was centrifuged at 7,000 rpm at 25˚C for 20 min. The supernatant was mixed with an equal volume of saturated ammonium sulfate solution and placed on ice for 30 min. The mixture was centrifuged at 12,000 rpm at 4˚C for 30 min. The supernatant was discarded, and the pellet was suspended with 19 mL PBS by vortexing. The suspension was transferred to an ultracentrifuge tube (Quick-Seal Polypropylene Tube 25x89 mm; Beckman Coulter, Indianapolis, IN, USA). Subsequently, OptiPrep (Alere Technologies AS, Oslo, Norway) was added to the bottom of the tube using a needle. A gradient of OptiPrep was created using a density gradient generator (Gradient Master 108, BioComp Instruments, Fredericton, Canada) using the following settings: holder, SW28; cap, long; solute, opti; top and bottom percentages of the solute, 5–20%; units, w/w; time, 3 min; angle, 80˚; and speed, 20 rpm. The tube was centrifuged (Optima XPN-80, Beckman Coulter) with a vertical rotor (VTi-50, Beckman Coulter) at 50,000 rpm at 16˚C for 16 hours. The tube was transferred to a fractionation unit (Fraction recovery system, 270-331580, Beckman Coulter). The bottom of the tube was punctured, and the solution was aliquoted sequentially. One μL aliquots from the fractions containing AAV were diluted into 999 μL VDB. The solutions were mixed with 3 μL proteinase K (10 mg/mL) and incubated at 52˚C for 45 min. The solutions were then incubated at 95˚C for 10 min to inactivate proteinase K, then were stored at 4˚C. The solution was used for titration.

Virus vector injection

Mice were anesthetized with 1–2% isoflurane and fixed on a stereotaxic frame. The scalps were opened and the skull above the injection site was drilled. A glass pipette (GC150-10, Harvard Apparatus, Holliston, MA, USA) made with a puller (P-97, Sutter Instrument, Novato, CA, USA) was used for injection of AAV. In the bilateral PVN (in mm, AP −0.5 from the bregma, ML 0.5 from the midline, DV −4.2 from the brain surface), 600 μL/site of AAV solution was injected by air pressure pulses (30–140 kPa pressure, 50 msec duration, 1 Hz frequency, total 5–10 min/site) regulated by a Pneumatic Picopump (World Precision Instruments, Sarasota, FL, USA) with a pulse generator (SEN-

7103, Nihon Kohden, Tokyo, Japan). The injected animals were used for subsequent experiments at least 3 weeks after injection.

Fixed brain slices

Mice were deeply anesthetized with isoflurane and perfused with 25 mL chilled saline followed by 25 mL chilled 10% formalin. After decapitation, each skull was carefully removed and brains were placed into chilled 10% formalin for post-fixation overnight. After post-fixation, brains were placed into 30% sucrose containing PBS for cryoprotection for at least 48 hours. After cryoprotection, brains were placed into O.C.T. compound (Sakura Finetek Japan Co., Ltd., Tokyo, Japan) and frozen at −80˚C for 20 min then placed into a −20˚C cryostat (CM3050 S; Leica Biosystems, Nussloch, Germany). Embedded brains were fixed on a stage using O.C.T. compound and sliced at a thickness of 40 µm. Slices were stored in PBS with 0.05% NaN_3.

Cell counts

Every 1 in 4 cryo-sectioned brain slices were used for cell counts. Slices were mounted on slide glasses and were visualized and imaged using a confocal microscope (LSM710, Carl Zeiss, Oberkochen, Germany). Images were obtained of the YFP and tdTomato fluorescence from slices which expressed YFP at the first and second highest levels for each animal (n=7). Brain slices from 2 animals in which YFP was expressed in fewer than 10 cells in the ventral PVN were excluded from the cell counts. The number of cells positive for only YFP or both YFP and tdTomato was counted manually from 4 images/animal consisting of 2 left and 2 right hemispheres. tdTomato/YC and occupancy were first calculated from each image; the mean of the 4 images/animal is shown as the individual data for each animal in **Fig. 1d, e**.

Acute brain slices

Animals were anesthetized with isoflurane and decapitated. The brains were immediately removed from the head and incubated in ice-cold cutting solution. Brains were sliced at a thickness of 250 µm using a vibratome (VT1200S, Leica). The slices were incubated in aCSF at 35ºC for 1 hour then at room temperature for at least hour.

Substances for screening

The compounds used for screening are listed in **Table 1**. We used concentrations approximately 100-times higher than the expected dissociation constant (K_d) of each receptor: for small molecules including amines, amino acids and nucleic acids, in which K_d values are around 1 µM, we used 100 µM. For lipids, in which K_d values are around 10 nM, we used 1 µM. For peptides, in which K_d values are around 1 nM, we used 100 nM or 1 µM.

Calcium imaging

Brain slices were placed in a chamber (RC-26G, Warner Instruments, Hamden, CT, USA) perfused with aCSF at 1.5 mL/min using a rotary pump (Dynamax, Rainin, Mettler Toledo, Schweiz, Switzerland; or Miniplus3, Gilson, Middleton, WI, USA). The slice was anchored with a harp (SHD-26GH, Warner Instruments) to avoid movement. A microscope (BX51WI, Olympus, Tokyo, Japan) was equipped with two objective lenses (UMPlanFl 20x 0.50W and LUMPlanFl/IR 40x 0.80W, Olympus), a filter cube (U-MF2) with a dichroic mirror for CFP excitation (FF458-Di02-25×36, Semrock, Rochester, NY, USA), an optical splitter (W-VIEW GEMINI, Hamamatsu photonics, Hamamatsu, Japan) with band-pass emitters (FF01-483/32-25 and FF01-542/27-25, Semrock) and a dichroic mirror for YFP/CFP recording (FF509-FDi01-25×36, Semrock), an electron-multiplying charge-coupled device (EMCCD) camera (iXon Ultra 897 or iXon Ultra 888, Andor, Oxford Instruments, Abingdon, UK) and a light source (Spectra X, Lumencor, Beaverton, OR, USA). For excitation, blue light (440 ± 20 nm, 50–210 μW/mm^2, 100 msec) was applied. The camera was controlled (acquisition: 100 msec; transport speed: 17-bit, 17 MHz for iXon Ultra 897, 14-bit, 10 MHz for iXon Ultra 888; EM Gain: 100) by software (MetaFluor, Molecular Devices, San Jose, CA, USA) through TTL communication via a device (USB-6501, National Instruments, Austin, TX, USA). The fluorescent signals for CFP and YFP were observed and recorded using the software. During the experiments, the YFP/CFP ratio of several cell bodies was calculated and used to monitor the concentration of calcium online.

Electrophysiological recordings

A glass pipette was made from a glass capillary (GC150-10, Harvard Apparatus) using a puller (P-1000, Sutter Instrument) to have a pipette resistance of 4–10 MΩ. KCl-based pipette solution was used as the internal solution. For patch-clamp recordings, an amplifier (Axopatch 200B, Molecular Devices) and a digitizer (Axon Digidata 1550A, Molecular Devices) were used. After identifying a cell expressing YC using a procedure similar to that for calcium imaging, the cell was contacted and ruptured with a glass pipette and maintained in a whole-cell current clamp mode. Negative current was injected to suppress spontaneous firing. Once the resting membrane potential was stable for >30 sec, command current (300 pA, 5 msec) was injected with a specific frequency (1, 2, 5 and 10 Hz) for 10 sec sequentially with a gap of more than 1 min between each frequency. Data was acquired using software (Clampex 10.7, Molecular Devices). During the electrophysiological recording, calcium imaging of the recorded cell was also performed.

Substance screening

To monitor cell autonomous effects and to suppress the effects of synaptic inputs from other neurons,

the voltage-gated sodium channel blocker tetrodotoxin (1 μM) was added to the aCSF. For a single brain slice, 10 substances, at most, were screened sequentially. Each candidate substance was dissolved in aCSF. The solution was then applied for 2 min via perfusion. The "onset" of application was defined as the time when the substance solution started to flow into the chamber containing a brain slice. The "offset" of application was defined as the time when fresh aCSF started to flow into the chamber after substance application. During and after perfusion, calcium signals were monitored. The onset of application was set at more than 5 min after the last signal disturbance including focal adjustment and application of the substance. When the calcium signal was stable for more than 5 min after the offset of an application, the focus was adjusted or the next substance was applied. When any change in calcium signal was observed, imaging was continued until the signal returned to baseline and became stable for an additional 5 min. The "session" was defined as the recording between the onset of application and just before the timing of the next substance application or focus change. As controls, for detection of baseline, increased and decreased calcium concentrations, aCSF, glutamate and GABA were applied, respectively. For confirmation of cell survival, aCSF containing 40 mM KCl was applied at the end of recording. Each substance was examined at least twice in different orders in multiple slices from different animals.

Antagonist experiments

To examine the effects of an antagonist, the antagonist was applied for at least 3 min before the onset of the subject substance application. The subject substance was diluted into the antagonist solution and applied for 2 min. The antagonist was further applied for at least 5 min after the offset of substance application. After antagonist application, normal aCSF was perfused.

Analysis

Images of YFP and CFP were motion corrected and aligned if needed using an original program based on the Scale-Invariant Feature Transform[2] written in MATLAB (R2019a, MathWorks, Natick, MA, USA). Regions of interest (ROIs) were drawn to surround cell bodies. ROIs that included two or more cells, or cells that disappeared before the end of an experiment were omitted. The intensity of YFP and CFP was measured by Fiji[3], and subsequent calculations including the YFP/CFP ratio (Y/C ratio) were performed in MATLAB.

For patch-clamp recordings, the raw Y/C ratio was used for analysis. The peak Y/C ratio during current injection of each stimulation was subtracted by the mean Y/C ratio during the period 30 sec before current injection (R_0) to obtain the peak ΔR. ΔR was divided by R_0 to obtain the peak $\Delta R/R_0$.

For screening and antagonist experiments, the raw value of the Y/C ratio was corrected ($R_{correct}$) using the mean value of the raw Y/C ratio during the period 2 min before the onset of

substance application (R_{before}), the same as that during the period 2 min before the end of the "session" (R_{end}) by the calculation ($R_{correct}(t) = R(t) + t \times (R_{end} - R_{before}) / (t_{end} - t_{before})$, where t is time from the onset in sec, t_{end} is 1 min before the end of the "session", t_{before} is 1 min before the onset of a substance application and $R(t)$ is the raw Y/C ratio at t. $R_{correct}$ during the period 2 min before substance application was used as a baseline to calculate mean (μ) and standard deviation (σ) to obtain a Z-score ($\text{Z-score}(t) = [R_{correct}(t) - \mu]/\sigma$).

For screening, the mean Z-score during the 5 minutes after the onset of each substance application was used. Quartiles of the mean Z-score for each substance were calculated from the combined screening experiments. When the third quartile was greater than 2, the substance was defined as increasing $[Ca^{2+}]_i$; conversely, when the first quartile was less than −1, the substance was defined as decreasing $[Ca^{2+}]_i$. These definitions were established to conservatively detect a clear calcium change based on the mean Z-scores observed after application of glutamate and GABA.

For antagonist experiments, the integral of the Z-score during the 5 minutes after onset of a subject substance application was calculated for both the 1st (substance only) and 2nd (with or without antagonist) sessions (Z_{1st} and Z_{2nd}, respectively). Z_{2nd} was divided by Z_{1st} to obtain the 2nd/1st ratio for control and antagonist effects.

Statistics

Statistical analysis was performed using software (OriginPro, Version 2019, OriginLab Corporation, Northampton, MA, USA) and R (https://www.R-project.org/). A p-value less than 0.05 was considered statistically significant. To test normality, the Shapiro-Wilk test was performed. When data did not show normality, to test differences in distribution between control and subject compounds, the Mann-Whitney *U* test was performed (**Fig. 3f, 4f)**. To test population differences, a Kruskal-Wallis test followed by Dunn's test was performed (**Fig. 1h, 5f**).

1 Itoi, K. *et al.* Visualization of Corticotropin-Releasing Factor Neurons by Fluorescent Proteins in the Mouse Brain and Characterization of Labeled Neurons in the Paraventricular Nucleus of the Hypothalamus. *Endocrinology* **155**, 4054-4060, doi:10.1210/en.2014-1182 (2014).

2 Lowe, D. G. Distinctive Image Features from Scale-Invariant Keypoints. *International Journal of Computer Vision* **60**, 91-110, doi:10.1023/b:Visi.0000029664.99615.94 (2004).

3 Schindelin, J. *et al.* Fiji: an open-source platform for biological-image analysis. *Nature Methods* **9**, 676-682, doi:10.1038/nmeth.2019 (2012).

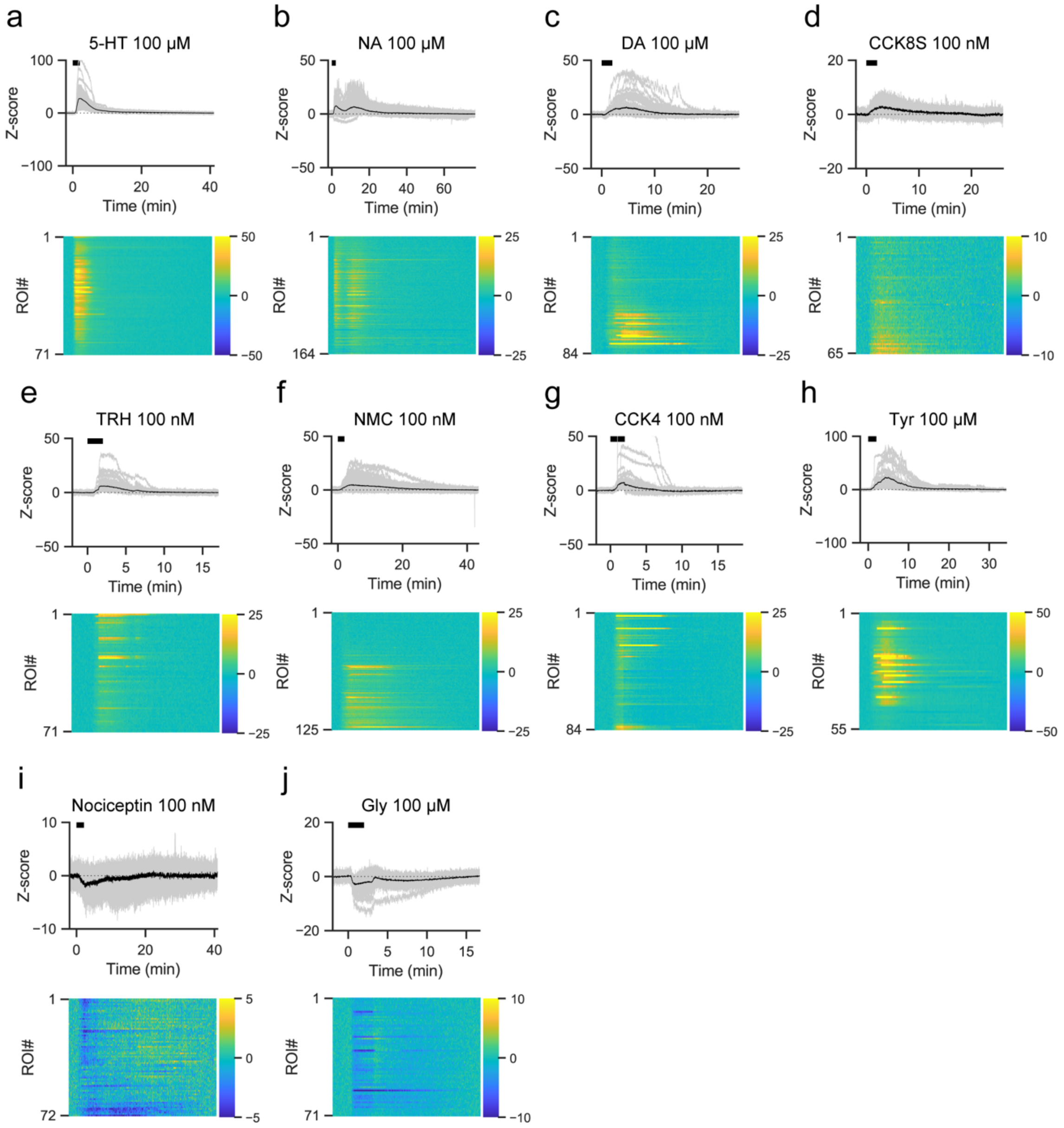

Supplementary Figure S1　Calcium signal change induced by each substance

(a–j) Z-scores of the YFP/CFP ratio recorded in a representative experiment during substance screening. Upper graphs show traces of individual ROIs (gray) and mean values (black). Black bars above the graphs indicate the application timing (2 min) of each substance (**a**, 5HT, serotonin; **b**, NA, noradrenaline; **c**, DA, dopamine; **d**, CCK8s, sulfated cholecystokinin octapeptide; **e**, TRH, thyrotropin-releasing hormone; **f**, NMC, neuromedin C; **g**, CCK4, cholecystokinin tetrapeptide; **h**, Tyr, tyramine; **i**, nociceptin; **j**, Gly, glycine. Heat maps show the Z-scores of individual ROIs, as indicated in the color bars at right.

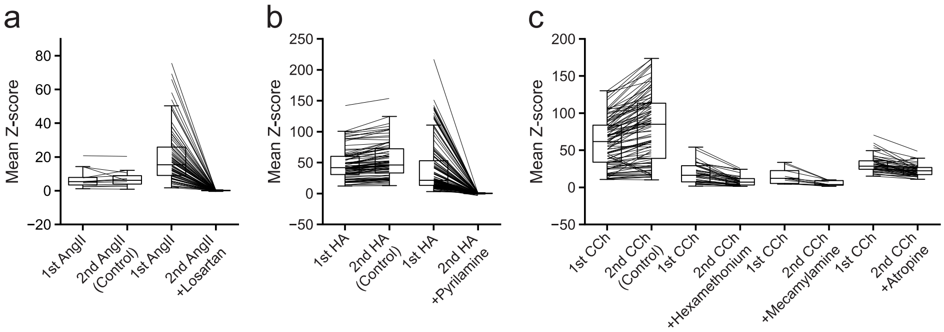

Supplementary Figure S2 Mean Z-scores in the antagonist experiments

(a–c) Box plots of the calcium signal changes induced by angiotensin II (AngII, **a**), histamine (HA, **b**) or carbachol (CCh, **c**) in the antagonist experiments. Lines between the 1st and 2nd box plots of each substance indicate the individual mean Z-scores of identical ROIs.

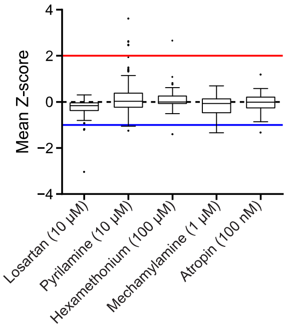

Supplementary Figure S3 Mean Z-scores during antagonist application

Box plots of calcium signal changes induced by the antagonists indicated below the graph. Red and blue lines indicate where the Z-score = 2 and −1, respectively. Dots indicate outliers.

Substance	Abbreviation	Concentration	# of cells	# of animals
Serotonin (5-hydroxytriptamine)	5HT	100 µM	494	6 (m1/f5)
Adrenocorticotropic hormone	ACTH	100 nM	279	3 (m1/f2)
Adenosine	Ade	100 µM	153	2 (f3)
Agouti-related peptide	AgRP	100 nM	118	2 (m2)
Alpha-melanocyte-stimulating hormone	aMSH	100 nM	166	2 (m1/f1)
Angiotensin II	Ang2	100 nM	125	2 (m1/f1)
Apelin	Ape	100 nM	121	2 (f2)
Adenosine triphosphate	ATP	100 µM	153	2 (f2)
Arginine vasopressin	AVP	100 nM	140	2 (m1/f1)
Cocaine- and amphetamine-related transcript	CART	100 nM	124	2 (m1/f1)
Carbachol	CCh	100 µM	239	3 (f3)
Cholecystokinin tetrapeptide	CCK4	100 nM	149	2 (m2)
Sulfated cholecystokinin octapeptide	CCK8S	100 nM	149	2 (m2)
Carcitonin gene-related peptide	CGRP	100 nM	121	2 (f2)
Cortisol	CORT	100 nM	282	3 (m1/f2)
Corticotropin releasing factor	CRF	100 nM	402	5 (m1/f4)
Dopamine	DA	100 µM	155	2 (m1/f1)
Dynorphin A	Dyn	100 nM	171	2 (f2)
Endothelin-3	EDT3	100 nM	140	2 (m2)
Gamma-aminobutyric acid	GABA	100 µM	1935	26 (m6/f20)
Galanin	Galanin	100 nM	193	3 (m3)
Growth hormone	GH	1 µM	253	2 (f2)
Ghrelin	Ghrelin	100 nM	212	3 (m3)
Glucagon-like peptide-1	GLP1	100 nM	171	2 (f2)
Glutamate	Glu	100 µM	1935	26 (m6/f20)
Glycine	Gly	100 µM	176	3 (f3)
Gastrin-releasing peptide	GRP	100 nM	601	7 (f7)
Histamine	HA	100 µM	224	2 (f2)
Interleukin-1β	IL-1β	100 nM	143	2 (f2)
Insulin	Insulin	100 nM	114	2 (f2)
Kisspeptin	Kiss	100 nM	134	2 (f2)
Leptin	Leptin	100 nM	130	2 (m1/f1)
Melanin-concentrating hormone	MCH	100 nM	126	2 (m1/f1)
Melatonin	MLTN	100 µM	108	2 (m1/f1)
Noradrenaline	NA	100 µM	662	8 (f8)
Neuromedin B	NMB	100 nM	134	2 (f2)
Neuromedin C	NMC	100 nM	188	2 (f2)
Neuromedin U	NMU	100 nM	140	2 (m1/f1)
Nociceptin	Nociceptin	100 nM	125	2 (m1/f1)
Neuropeptide B	NPB	100 nM	153	2 (f2)
Neuropeptide S	NPS	1 µM	128	2 (m2)
Neuropeptide W	NPW	1 µM	140	2 (m1/f1)
Neuropeptide Y	NPY	100 nM	140	2 (m1/f1)
Obestatin	Obestatin	100 nM	127	2 (f2)
Orexin-A	ORX-A	100 nM	140	2 (m1/f1)
Orexin-B	ORX-B	100 nM	179	3 (m1/f2)
Oxytocin	OXT	100 nM	140	2 (m1/f1)
Pituitary adenylate cyclase-activating polypeptide	PACAP27	100 nM	156	2 (m1/f1)
Prostaglandin D2	PGD2	1 µM	130	2 (m1/f1)
Prostaglandin E2	PGE2	1 µM	102	2 (m1/f1)
Prokineticin-2	PK2	100 nM	118	2 (m2)
Pancreatic polypeptide	PP	100 nM	100	2 (f2)
Prolactin-releasing peptide	PrRP	100 nM	171	2 (f2)
Peptide YY	PYY	100 nM	100	2 (f2)
Pyroglutamylated RF amide peptide	QRFP	100 nM	283	4 (m2/f2)
Secretin	Secretin	100 nM	125	2 (m1/f1)
Substance P	SP	100 nM	121	2 (f2)
Somatostatin	SST	100 nM	100	2 (f2)
Stresscopin	Stresscopin	100 nM	132	2 (f2)
Thyrotropin-releasing hormone	TRH	100 nM	169	3 (f3)
Tyramine	Tyr	100 µM	171	3 (f3)
Urocortin-3	UCN	100 nM	98	2 (f2)
Vasoactive intestinal peptide	VIP	100 nM	100	2 (f2)
Total			1935	26 (m6/f20)

Supplementary Table S1   Number of cells and animals used for substance screening

The number of cells and animals used for substance screening is shown in the table listed by substance (1st column), abbreviation (2nd column), concentration (3rd column), the number of cells (4th column) and the number of animals with their sex in parentheses (m, male; f, female; 5th column). The number of brain slices used for each substance was the same as the number of animals (1 slice per 1 animal).

**Substance / Antagonist**	**# of cells in vPVN**	**# of cells in dPVN**	**# of animals**
Angiotensin II / Control	12	52	1 (f1)
Angiotensin II / Losartan	77	30	2 (f2)
Histamine / Control	87	59	1 (f1)
Histamine / Pyrilamine	130	42	2 (m1/f1)
Carbachol / Control	63	98	2 (m1/f1)
Carbachol / Hexamethonium	42	40	2 (f2)
Carbachol / Mecamylamine	72	11	2 (m1/f1)
Carbachol / Atropin	47	48	1 (m1)

Supplementary Table S2 Number of cells and animals used for antagonist experiments

The number of cells and animals used for antagonist experiments is shown in the table listed by substance/antagonist (1st column), the number of cells in the vPVN (2nd column), the number of cells in the dPVN (3rd column) and the number of animals with their sex in parentheses (m, male; f, female; 4th column). The number of slices used for each experiment was the same as the number of animals (1 slice per 1 animal).

	**CRF01_2** (61/f)	**CRF02_1** (37/m)	**CRF03_1** (71/m)	**CRF04_1** (69/f)	**CRF05_1** (45/f)	**CRF06_2** (75/m)	**CRF07_1** (55/f)
1	Control	Control	Control	Control	Control	Control	Control
2	CRF (−7)	ACTH (−7)	NMU (−7)	OXT (−7)	PYY (−7)	Control [2]	Gly (−4)
3	GABA (−4)	MLTN (−4)	MCH (−7)	NPY (−7)	SST (−7)	PGD2 (−6)	Leptin (−7)
4	QRFP (−7)	CRF (−7)	NPW (−6)	NMU (−7)	VIP (−7)	CRF (−7)	MCH (−7)
5	5HT (−4)	TRH (−7)	ORX-A (−7)	AVP (−7)	PP (−7)	Leptin (−7)	PP (−7)
6	TRH (−7)	PGE2 (−6)	NPY (−7)	Insulin (−7)	Tyr (−4)	Ang2 (−7)	PYY (−7)
7	UCN (−7)	UCN (−7)	MLTN (−4)	ORX-A (−7)	Insulin (−7)	GABA (−4)	PGD2 (−6)
8	Stresscopin (−7)	ORX-B (−7)	ORX-B (−7)	CCh (−4)	GABA (−4)	Glu (−4)	Tyr (−4)
9	PGE2 (−7)	NA (−4)	AVP (−7)	NPW (−6)	Glu (−4)	HighK	Obestatin (−7)
10	NA (−6)	GABA (−4)	ACTH (−7)	NPW (−5)	HighK		SST (−7)
11	IL-1β (−7)	Glu (−4)	OXT (−7)	NA (−5)			VIP (−7)
12	GABA (−4) [2]	HighK	GABA (−4)	GABA (−4)			GABA (−4)
13	Glu (−4)		Glu (−4)	Glu (−4)			Glu (−4)
14	HighK		HighK	HighK			HighK

	**CRF08_1** (50/f)	**CRF09_1** (65/f)	**CRF10_1** (75/m)	**CRF11_1** (84/m)	**CRF12_1** (53/m)	**CRF13_1** (72/f)	**CRF14_1** (99/f)
1	Control	Control	Control	Control	Control	Control	Control
2	Gly (−4)	QRFP (−7)	QRFP (−7)	DA (−5)	NPS (−6)	PACAP27 (−7)	GRP (−7)
3	Ang2 (−7)	PK2 (−7)	L-NAME (−4)	DA (−4)	AgRP (−7)	PrRP (−7)	NMB (−7)
4	GABA (−4)	CCK8S (−7)	EDT3 (−7)	Ghrelin (−7)	Galanin (−7)	Obestatin (−7)	GLP1 (−7)
5	Glu (−4)	EDT3 (−7)	NPS (−6)	CCK4 (−7)	Ghrelin (−7)	Baclofen (−5)	AL-ORX-B (−7)
6	HighK	L-NAME (−4)	Galanin (−7)	CCK8S (−7)	Nociceptin (−7)	Adenosine (−5)	HA (−4)
7		AgRP (−7)	Baclofen (−5)	aMSH (−7)	PK2 (−7)	Dyn (−7)	CCh (−4)
8		EDT3 (−7)	Ghrelin (−7)	PACAP27 (−7)	CART (−7)	CORT (−6)	PrRP (−7)
9		PGE2 (−6)	GABA (−4)	5HT (−4)	Secretin (−7)	Nociceptin (−7)	Dyn (−7)
10		CCK4 (−7)	Glu (−4)	GABA (−4)	CORT (−7)	Secretin (−7)	GABA (−4)
11		Galanin (−7)	HighK	Glu (−4)	CORT (−6)	GLP1 (−7)	Glu (−4)
12		GABA (−4)		HighK	GABA (−4)	GABA (−4)	HighK
13		Glu (−4)			Glu (−4)	Glu (−4)	
14		HighK			HighK	HighK	

	**CRF15_1** (82/f)	**CRF16_1** (63/f)	**CRF17_1** (58/f)	**CRF20_3** (71/f)	**CRF21_1** (125/f)	**CRF22_1** (34/f)	**CRF51_1** (60/f)
1	Control	Control	Control	Control	Control	Control	Control
2	AL-ORX-B (−7)	Kiss (−7)	SP (−7)	Gly (−4)	NMC (−7)	NA (−4)	GRP (−7)
3	NPB (−7)	NMB (−7)	CGRP (−7)	GRP (−7)	HA (−4)	Tyr (−3)	5HT (−4)
4	GRP (−7)	CGRP (−7)	Ape (−7)	Tyr (−4)	GABA (−4)	GABA (−4)	NA (−4)
5	QRFP (−7)	SP (−7)	GABA (−4)	ORX-B (−7)	Glu (−4)	Glu (−4)	GABA (−4)
6	aMSH (−7)	Ape (−7)	CORT (−7)	TRH (−7)	HighK	HighK	Glu (−4)
7	Adenosine (−4)	NMC (−7)	CRF (−7)	NA (−4)			
8	GH (−6)	SP (−7) [2]	Glu (−4)	ATP (−4)			
9	ATP (−4)	GABA (−4)	HighK	GABA (−4)			
10	IL-1β (−7)	Glu (−4)		Glu (−4)			
11	GABA (−4)	HighK		HighK			
12	Glu (−4)						
13	HighK						
14							

	**CRF52_1** (71/f)	**CRF53_1** (164/f)	**CRF54_1** (54/f)	**CRF57_1** (71/f)	**CRF58_1** (171/f)
1	Control	Control	Control	Control	Control
2	5HT (−4)	NA (−4)	GRP (−7)	CART (−7)	GH (−6)
3	NA (−4)	GRP (−7)	NA (−4)	GH (−7)	CRF (−7)
4	GRP (−7)	5HT (−4)	5HT (−4)	Kiss (−7)	ACTH (−7)
5	GABA (−4)	GABA (−4)	GABA (−4)	NMB (−7)	CORT (−7)
6	Glu (−4)	Glu (−4)	Glu (−4)	NPB (−7)	NA (−4)
7				Stresscopin (−7)	GABA (−4)
8				Adenosine (−4)	Glu (−4)
9				DA (−4)	HighK
10				Control [2]	
11				CCh (−4)	
12				GABA (−4)	
13				Glu (−4)	
14				HighK	

Supplementary Table S3 Order of substances used for screening

The order of substances used for screening is shown in the table. The 1st row shows the ID of the experiment and the number of ROIs with the sex of the animals (m, male; f, female) in parentheses (ROIs/sex). The 2nd to 15th rows show the substances with concentrations in parentheses. Control indicates aCSF without added substances. Abbreviations are listed in Table 1, except for the following: HighK, high concentration of potassium ions; L-NAME, N omega-Nitro-L-arginine methyl ester; AL-ORX-B, [Ala11, d-Leu15]orexin-B; [2], second trial in the same slice. Each column shows an individual experiment. Substance names shown in blue text are the substances used for screening. Data for substances highlighted by gray rectangles were not used in this study.

	Angiotensin II / Control	Angiotensin II / Losartan	
	**CRF43_2** (v12/d52/f)	**CRF41_1** (v39/d8/f)	**CRF42_2** (v38/d22/f)
1	Control	Control	Control
2	AngII (−7)	AngII (−7)	AngII (−7)
3	AngII (−7) [2]	LP (−5) & AngII (−7)	LP (−5) & AngII (−7)

	Histamine / Control	Histamine / Pyrilamine	
	**CRF32_1** (v87/d58/f)	**CRF33_1** (v57/d0/m)	**CRF34_1** (v73/d42/f)
1	Control	Control	Control
2	HA (−5)	HA (−5)	HA (−5)
3	HA (−5) [2]	PM (−6) & HA (−5)	PM (−6) & HA (−5)

	Carbachol / Control		Carbachol / Hexamethonium	
	**CRF35_1** (v32/d50/m)	**CRF46_2** (v31/d48/f)	**CRF46_1** (v25/d23/f)	**CRF47_1** (v17/d17/f)
1	Control	Control	Control	Control
2	CCh (−4)	CCh (−4)	CCh (−4)	CCh (−4)
3	CCh (−4) [2]	CCh (−4) [2]	HEX (−4) & CCh (−4)	HEX (−4) & CCh (−4)

	Carbachol / Mecamylamine		Carbachol / Atropin
	**CRF42_1** (v37/d6/f)	**CRF45_1** (v35/d5/m)	**CRF36_1** (v47/d48/m)
1	Control	Control	Control
2	CCh (−4)	CCh (−4)	CCh (−4)
3	MEC (−6) & CCh (−4)	MEC (−6) & CCh (−4)	ATR (−7) & CCh (−4)

Supplementary Table S4 Order of substances used for antagonist experiments

The order of substances used for antagonist experiments is shown in the table. The first row describes the experimental condition (substance/antagonist). The 2nd row shows the experimental ID and the number of ROIs in the vPVN (v) and dPVN (d), with the sex of the animals (m, male; f, female) shown in parentheses (vPVN/dPVN/sex). The 3rd to 5th rows show the substances with concentrations in parentheses. Control indicates aCSF without added substances. Abbreviations are listed in Table 1, except for the following: LP, losartan; PM, pyrilamine; HEX, hexamethonium; MEC, mecamylamine; ATR, atropine; [2], second application in the same slice. Each column shows an individual experiment. Data for the controls shown highlighted by gray rectangles were not used in this study.
